# Community collectivization and its association with consistent condom use and STI treatment-seeking behaviors among female sex workers and high-risk men who have sex with men/transgenders in Andhra Pradesh, India

**DOI:** 10.1080/09540121.2012.749334

**Published:** 2013-06-09

**Authors:** Niranjan Saggurti, Ram Manohar Mishra, Laxminarayana Proddutoor, Saroj Tucker, Dolly Kovvali, Prabhakar Parimi, Tisha Wheeler

**Affiliations:** a HIV and AIDS Program, Population Council, New Delhi, India; b Hindustan Latex Family Planning Promotion Trust, Hyderabad, India; c India HIV/AIDS Alliance, Hyderabad, India; d Bill & Melinda Gates Foundation, Washington, DC, USA

**Keywords:** community mobilization, community collectivization, condom use, female sex workers, men who have sex with men/transgenders

## Abstract

We examine community collectivization among female sex workers (FSWs) and high-risk men who have sex with men and transgenders (HR-MSM) following several years of HIV prevention programming with these populations, and its association with selected outcome indicators measuring individual behaviors (condom use with different partners and sexually transmitted infection [STI] treatment-seeking from government health facilities). Data for this study were collected from a large-scale cross-sectional survey conducted in 2010–2011 among FSWs (sample size: 3557) and HR-MSM (sample size: 2399) in Andhra Pradesh, India. We measured collectivization among FSWs in terms of three binary (low, high) indices of collective efficacy, collective agency, and collective action. Collectivization among HR-MSM was measured by participation in a public event (no, yes), and a binary (low, high) index of collective efficacy. Adjusted odds ratios (adjusted OR) and their 95% confidence intervals (CI) were computed to assess the relationships between collectivization and outcome indicators directly and through mediation of variables such as self-efficacy for condom use and utilization of government health facilities. Results show that among FSWs, high levels of collective efficacy (adjusted OR: 1.3, 95% CI: 1.1–1.7) and collective action (adjusted OR:1.3, 95% CI: 1.1–1.8) were associated with consistent condom use (CCU) with regular clients. Among HR-MSM, participation in a public event (adjusted OR: 2.7, 95% CI: 2.0–3.6) and collective efficacy (adjusted OR: 1.9, 95% CI: 1.5–2.3) were correlated with condom use with paying partners. The association between collectivization and outcome indicators continued to be significant in most cases even after adjusting for the potential mediators. Indicators of collectivization exhibited significant positive association with self-efficacy for condom use and service utilization from government health facilities among both FSWs and HR-MSM. The association of high levels of collectivization with CCU, STI treatment- seeking from government health facilities, ability to negotiate for condom use, and self-efficacy in utilizing government health facilities is relevant to effort to improve the effectiveness and sustainability of HIV prevention programs in India and beyond.

## Introduction

Over the past decade, studies across the world have indicated that peer-led targeted HIV prevention interventions result in increased HIV knowledge (Ford, Wirawan, Reed, Muliawan, & Wolfe, 2002) and condom use (Blanchard et al., 2005; Halli, Ramesh, O'Neil, Moses, & Blanchard, 2006; Rou et al., 2007; Walden, Mwangulube, & Makhumula-Nkhoma, 1999) as well as decreased prevalence of sexually transmitted infections (STIs) (Ford et al., 2002; Rou et al., 2007) and HIV (Ghys et al., 2001). It is recognized also that peer-led interventions that seek to change behavior require support through the development of “health-enabling social environments” (Foster, 1990; Jana & Singh, 1995; Tawil, Verster, & O'Reilly, 1995). Increasingly, and in particular for those most at risk of acquiring HIV (key populations), studies recommend that HIV prevention programs must go beyond peer-led intervention approaches to address structural drivers – the complex social, cultural, political, and economic vulnerabilities faced by those who are most marginalized. (Blanchard et al., 2005; Chattopadhyay & McKaig, 2004; de Souza, 2009; Halli et al., 2006; Hays, Rebchook, & Kegeles, 2003; Nath, 2000; Parker, Easton, & Klein, 2000). Building upon lessons from micro-level community mobilization as a part of structural interventions in HIV risk reduction (Jana & Singh, 1995; Latkin & Knowlton, 2005; Nath, 2000), Avahan, the India AIDS Initiative, launched a large-scale HIV prevention intervention in 2003 with key populations across six high-HIV prevalence states in India (Avahan, 2008). One of its goals was to mobilize communities of female sex workers (FSWs) and high-risk men who have sex with men/transgenders (HR-MSM) to manage and implement prevention programs themselves (Avahan, 2008; Chandrasekaran et al., 2006).

Community mobilization has been defined and operationalized in numerous ways and incorporates concepts from a range of approaches including social policy (Kothari, 2001), participatory development (Chambers, 1997), and structural interventions (Blankenship, Friedman, Dworkin, & Mantell, 2006). Avahan describes community mobilization as the process by which key population members “utilize their intimate knowledge of vulnerability to overcome the barriers they face and realize reduced HIV risk and greater self-reliance through their collective action” (Avahan, 2008). Thus, Avahan's community mobilization interventions seek to address HIV risk in key populations by fostering community-level changes that may alter power relations between marginalized and dominant groups (Blankenship et al., 2006; Galavotti et al., 2012). One of the earliest examples of community mobilization strategies in India, the Sonagachi Project's community-led structural interventions (Jana, Basu, Rotheram-Borus, & Newman, 2004), was a major contributor to Avahan's approach to community mobilization (Galavotti et al., 2012). Although most of Avahan's interventions were standardized across states and districts, the implementation of community mobilization varied, with the differences mainly in group structures and focus on the local priorities and needs of communities (Chakravarthy, Joseph, Pelto, & Kovvali, 2012; Gaikwad, Bhende, Nidhi, Saggurti, & Ranebennur, 2012; Punyam et al., 2012). Community mobilization (collectivization) aimed not only to empower key populations as a group for vulnerability reduction, but also to enhance their self-efficacy (defined as ability to control and make decisions about one's own behaviors) which in turn influences the adoption and maintenance of healthy behaviors (Bandura, 1977; Galavotti et al., 2012; Wheeler et al., 2012).

While the importance of community mobilization is increasingly recognized, more data are needed to understand how it contributes to the HIV/AIDS response in India, particularly in increasing knowledge, promoting safer sexual behavior (Blankenship, West, Kershaw, & Biradavolu, 2008; Cornish & Ghosh, 2007; Halli et al., 2006; Jana & Singh, 1995), and influencing self-efficacy of key populations. This paper describes an analysis of the degree of collectivization among FSWs and HR-MSM in Andhra Pradesh state in southern India, and examines its relationship with individuals’ condom use behavior, utilization of government health centers for STI treatment, self-efficacy for condom use with commercial partners, and self- efficacy for service utilization from government health facilities. Furthermore, we examine the mediating effects of self-efficacy on condom use with clients and STI treatment from government health facilities, by the degree of collectivization.

## Methods

This paper utilizes data from the Behavioral Tracking Survey, a cross-sectional behavioral survey conducted during 2010–2011 among FSWs and HR-MSM in Andhra Pradesh to monitor key components of the Avahan program: community mobilization, safe sex behavior, and STI treatment-seeking behavior. FSWs were recruited for the survey from nine program districts and HR-MSM from six program districts from a total of 23 districts where the Avahan program implemented the HIV prevention program with both FSWs and HR-MSM.

Survey districts were selected to include areas where the HIV prevention program was being implemented but where no surveys specifically measuring community mobilization had previously been conducted. For both groups (FSWs and HR-MSM), a sample size of 400 completed interviews was calculated for each district based on prevalence of consistent condom use (CCU) and expected level of change with each unit change in the degree of community mobilization.

To prepare the sampling frame for the selection of FSWs from each hot spot (place where FSWs congregate to solicit clients), a rapid mapping exercise was conducted using key informant interviews with local community members, police staff, and social workers. This validated the existing list of hot spots originally developed by the program-implementing agency. The hot spots were then grouped into two categories: (1) non-public (brothels, hotels, lodges, roadside cafes, and homes) and (2) public (streets, market areas, highways, and cinemas). A probability sampling method was used to select respondents. Conventional cluster sampling was used for nonpublic hot spots and time-location cluster (TLC) sampling for public hot spots (Magnani, Sabin, Saidel, & Heckathorn, 2005; Saidel et al., 2008). The TLC method involved dividing a hot spot into several clusters based on the time slots (e.g., 5 pm–9 pm) when FSWs and HR-MSM congregated at the hot spot and then selecting the required number of clusters randomly. In the second stage, respondents were randomly selected within each selected hot spot.

From a total of 5192 listed FSWs from selected sampling sites, 4240 FSWs were approached and 3557 agreed to participate in the survey, a participation rate of 83.9%. Of the 3546 estimated HR-MSM from the sites sampled, 2984 were approached and 2399 agreed to participate in the survey, a participation rate of 80.4%. This high response rate is attributed to the recruitment method, which drew upon the knowledge of community peer outreach workers. Reasons for nonparticipation among FSWs and HR-MSM included phone calls from prospective clients, interruptions for dialog with clients, heavy rain, and objections from pimps/brokers or madams.

All interviews were conducted by trained researchers with verbal and written skills in Telugu, the local language of Andhra Pradesh. The survey instrument was developed in English and translated into Telugu. The translated forms were reviewed by study investigators fluent in both languages. The interview schedule was pre-tested in communities similar to the survey sites. All the interviews were held in a private location specifically hired for the survey, or in a location convenient to the study participants. Field staff checked the data immediately after the interviews to ensure accuracy and completeness of the questionnaires. A user-written computer program in CSPro (version. 4.0) was used for double data entry by trained data entry officers.

The original behavioral tracking survey design and questionnaires were approved by the institutional review boards of Family Health International and the Karnataka Health Promotion Trust. Verbal consent was obtained from all respondents prior to participation in the interview. For ethical reasons, only those FSWs and HR-MSM who were at least 18 years of age were interviewed. No names and addresses were recorded on the questionnaires. Participants were not compensated but were referred to project services run by implementing agencies in the study districts.

### Measures

The sociodemographic variables adjusted for in the analysis were age (18–29 years, 30 +years); formal education (yes, no); marital status (never married, currently married, and formerly married); no source of income other than sex work (yes, no); duration of sex work (0–2 years, 2–4 years, 5–9 years, and 10 + years); mobility for sex work within and/or outside district in past two years (yes, no); currently in debt (yes, no); and place of solicitation for sex (brothels, homes, and public places). The independent variables used in the FSW analysis were collective efficacy, collective agency, and collective action; these were made up of multiple indicators comprising a composite index described in detail in Table 1. For the HR- MSM analysis, the same collective efficacy variable was used, as well as a single-indicator variable for participation in public events.

**Table 1.** Definition and coding of community mobilization indicators used in Behavioral Tracking Survey (2010–2011).

Community mobilization indicator	Definition and coding
Collective efficacy (both FSWs and HR-MSM)	Collective efficacy is the belief of the affected community in its power to work together to bring positive changes. It was measured based on responses to the question: How confident are you that FSWs in your community can work together to achieve the following goals: keep each other safe from harm; increase condom use with clients; speak up for your rights; and improve your lives? Responses to these questions included: not at all, somewhat, very, and completely confident. A composite index was constructed. The scale had reliability (Cronbach's alpha) of 0.831 for FSWs and 0.928 for HR-MSM. The index score was further divided into two equal categories of collective efficacy: low (1–2.4999) and high (2.5–4).
Collective agency (only for FSWs)	Collective agency is the choice, control, and powers that poor or marginalized groups have to act for themselves to claim their rights (whether civil, political, economic, social or cultural) and to hold others accountable for these rights. It was measured based on responses to the question: In the past 6 months, have you negotiated with or stood up against the following stakeholders (police, madam/broker, local goon [gang member], clients or any other sexual partner) in order to help a fellow sex worker or to help fellow sex workers? A separate question for each of the above stakeholders was asked, with the possible binary response categories “Yes” (coded as 1) and “No” (coded as 0). Using these four questions and corresponding responses, an index was constructed, with the scale values ranging from 0–1, which had reliability (Cronbach's alpha) of 0.782. The index score was further divided into two equal categories of collective agency: low (0–0.4999) and high (0.5–1).
Collective action (only for FSWs)	Collective action is the strategic and organized activities by mobilized community members to increase the community's visibility in wider society and present or enact its agenda for change (for example, through rallies, demonstrations, or meetings with stakeholders). It was measured based on responses to the following seven questions: Whether the sex workers group come together to demand/help for the following: (1) ration card, (2) voter card, (3) bank account, (4) free education for children, (5) health insurance, (6) representation in government forums, (7) better health services from the government. A separate question was asked for each of the above social entitlements and services with the possible binary response categories “Yes” (coded as 1) and “No” (coded as 0). Using these seven questions and corresponding responses, an index was constructed, with the scale values ranging from 0 to 1, which had reliability (Cronbach's alpha) of 0.782. The index score was further divided into two equal categories of collective action: low (0–0.4999) and high (0.5–1).
Participation in public events (only for HR-MSM)	Participation in public events was measured as whether the respondents participated in any public event in the past six months at risk of being identified as HR-MSM (no, yes). It was derived based on single questions in the questionnaire.

Notes: FSWs: Female sex workers; HR-MSM: High-risk men who have sex with men/transgenders.

The key outcome indicators used for analyses were (1) CCU with commercial sex partners and (2) STI treatment-seeking from government health facilities. While examining the association between the degree of collectivization and outcome indicators, we also assessed the role of individual-level efficacy variables as potential mediating factors for indirect benefits of community mobilization. We included (1) self-efficacy for condom use with commercial partners, as a potential mediator for relationship between collectivization and CCU with commercial partners and (2) self-efficacy for service utilization from government health facilities, as the potential mediator for relationship between collectivization and STI treatment-seeking from government health facilities (see Table 2 for definitions).

**Table 2.** Measurement of outcome indicators and mediators used in Behavioral Tracking Survey (2010–2011).

	Definitions and coding
*Outcome indicators*	
CCU with occasional clients and regular clients (only for FSWs)	CCU with a given type of clients (occasional, regular) was defined as use of condom in every sexual encounter with that type of client. Occasional clients were defined as men whom FSWs did not knew or do not recognize their faces. Regular clients were defined as men whom FSWs knew well and could recognized their faces.
CCU with paid partners and paying partners (only for HR-MSM)	CCU with a given type of partner (paid, paying) was defined as use of condom in every sexual encounter with that type of partner. Paid partners were defined as men/transgenders whom the respondent paid for sex. Paying partners were defined as men/transgenders who paid the respondent for sex.
STI treatment from government health facilities	The STI treatment-seeking from government health facilities in the past one year was defined as whether or not an individual visited government health facilities for treatment of any STI, irrespective of his/her seeking treatment for STIs at other places. Among FSWs, any STI was defined as presence of any of the following two symptoms in the past one year: (1) genital sore/ulcer; (2) yellowish/greenish discharge from vagina. Among HR-MSM, any STI was defined as presence of any of the following three symptoms in past one year: (1) genital sore/ulcer; (2) anal sore/ulcer; and (3) unusual discharge from penis/ anus.
*Potential mediators*	
Self-efficacy for condom use with commercial partners	Self-efficacy for condom use with commercial partners refers to the ability of FSWs/HR-MSM to negotiate for condom use with their commercial partners. It was measured by three questions assessing self-efficacy for each time condom use with commercial partners: How confident are you that you can use a condom with each commercial partner when (1) he gets angry with you; (2) he offers you more money for sex without a condom; or (3) you have been using alcohol or drugs? Responses to these questions included: not at all (coded as 1), somewhat (coded as 2), very (coded as 3), and completely confident (coded as 4). Using these four questions and corresponding responses, an index was constructed, with the scale value ranging from 1 to 4, which had reliability (Cronbach's alpha) of 0.850 for FSWs and 0.898 for HR-MSM. The index score was further divided into two equal categories of self-efficacy for condom use with clients: low (1–2.4999) and high (2.5–4).
Self-efficacy for service utilization from government health facilities	Self-efficacy for service utilization from government health facilities among FSWs/ HR-MSM was measured based on responses to the following two questions: (1) How confident are you that you can go to the government health clinic to get reproductive health services you need if the health workers there treat you badly; and (b) How confident are you that you can go to the government health clinic to get the reproductive health services even if the health worker knows that you are a FSW/MSM? Responses to these questions included: not at all (coded as 1), somewhat (coded as 2), very (coded as 3), and completely confident (coded as 4). Using responses to the two questions an index was constructed with the scale values ranging from 1 to 4 which had reliability (Cronbach's alpha) of 0.869 for FSWs and 0.927 for HR-MSM. The index score was further divided into two equal categories of self-efficacy for service utilization: low (1–2.4999) and high (2.5–4).

Notes: FSWs, female sex workers; HR-MSM, high-risk men who have sex with men/transgenders; STI, sexually transmitted infection; CCU, consistent condom use

### Data analysis

Descriptive statistics (i.e., means, standard deviations, and proportions) and bivariate analyses were presented to describe the strength and association of collectivization and the outcome indicators. Adjusted odds ratios (adjusted OR) and their 95% confidence intervals (CI) were estimated, adjusting for sociodemographic characteristics, to assess the independent relationships of degree of collectivization with the outcome indicators and potential mediators. A significant association between collectivization and outcome measures was considered to be the prerequisite for the mediation analyses (Baron & Kenny, 1986; Hayes, 2009). The effect of a collectivization indicator on any study outcome was considered to be mediated through a potential mediator if the following conditions were met (1) the collectivization indicator was significantly associated with study outcome, (2) collectivization was significantly associated with the potential mediator, and (3) the relationship between collectivization and the study outcome declined when the mediating variable was entered into the regression model (Baron & Kenny, 1986; Dingemans, Spinhoven, & van Furth, 2007; Hayes, 2009). The first two conditions were evaluated by assessing the independent relationships of collectivization indicators with the study outcomes and potential mediating variables. The third condition was evaluated by entering the potential mediating variable as one of the independent variables in the multivariable logistic regression models used to examine the relationships between collectivization and study outcomes. All analyses described above were conducted separately for FSWs and HR-MSM using STATA software (version 11.0).

## Results

### Female sex workers

Nearly half (47.2%) of the FSWs were aged 30 years or more (average age: 29.4 years), 42.4% had formal schooling, and three-fifths (60.6%) were currently married. The average duration of practicing sex work was 4.8 years (Table 3).

**Table 3.** Demographics, community mobilization indicators, direct and indirect outcome indicators among FSWs (*N* = 3557) and HR-MSM (*N* = 2399), Andhra Pradesh, India.

	FSWs		HR-MSM	
Background characteristics	*N*	Percentage or Mean (SD)	*N*	Percentage or Mean (SD)
Sociodemographic				
Age ≥30 years	1679	47.2	763	31.8
Average age (years)		29.4 (5.7)		27.6 (6.1)
Formal schooling^a^	1510	42.4	1780	74.2
Currently married	2154	60.6	779	32.5
Formerly married^b^	1125	31.6	89	3.7
No source of income other than sex work	773	21.7	302	12.6
Currently in debt	2844	79.9	976	40.7
Mobility for sex work				
Visited places and had sex in past two years	1410	39.6	1852	77.2
Visited places outside district and had sex in past two years	996	28.0	1428	59.5
Duration of practicing sex work (for FSWs)/anal sex with man/transgenders (for MSM) (in years)				
< 2	243	6.8	60	2.5
2–4	1645	46.2	464	19.3
5–9	1332	37.5	851	35.5
10 or more	337	9.5	1024	42.7
Average duration		4.8 (3.3)		9.2 (5.9)
Place of solicitation for sex work				
Home	1124	31.6		
Public places (streets, highways, and parks)	2233	62.8		
Brothels or lodges	200	5.6		
Community mobilization indicators				
Collective efficacy: high	2815	79.1	1489	62.1
ollective agency: high	1339	37.7		
Collective action: high	663	18.6		
Participation in public event in past six months			1916	79.9
Outcome indicators				
CCU with occasional clients	2691	75.7		
CCU with regular clients^c^ (*N* = 3521)	2380	67.6		
CCU with paid partners (*N* = 620)			433	69.8
CCU with paying partners^e^ (*N*=1679)			1213	72.2
STI treatment from government health facilities in past one year (FSWs: *N* = 1521; HR-MSM: *N* = 320)	873	57.4	132	41.3
Potential mediators				
Self efficacy for condom use with clients: high	2481	69.7	1731	72.2
Self efficacy for service utilization from government health facilities: high	1978	55.6	1323	55.2

Notes: Average refers to the mean values.

FSWs, female sex workers; HR-MSM, high-risk men who have sex with men/transgenders; STI, sexually transmitted infection; CCU, consistent condom use; SD, standard deviation.

a Formal schooling refers to the ability to both read and write.

b Formerly married refers to those who were divorced, separated, or widowed.

c Among FSWs who reported having regular clients in past one year.

d Among HR-MSM who reported having paid partners (partners whom respondents paid for sex) in past one year.

e Among HR-MSM who reported having paying partners (partners who paid respondents for sex) in past one year.

f Among those FSWs and HR-MSM who reported having any STI in past one year. Among FSWs, any STI was defined as presence of any of the following two symptoms in the past one year: (1) genital sore/ulcer; (2) yellowish/greenish discharge from vagina. Among HR-MSM, any STI was defined as presence of any of the following three symptoms in past one year: (1) genital sore/ulcer; (2) anal sore/ulcer; and (3) unusual discharge from penis/anus.

About two-fifths (39.6%) reported visiting other places for sex work. The large majority of FSWs (62.8%) solicited clients from public places and homes (31.6%). About four-fifths (79.1%) reported a high degree of collective efficacy, whereas the percentages for collective agency and collective action were 37.7% and 18.6%, respectively.

FSWs’ high degree of collectivization exhibited significant positive association with most outcome indicators and potential mediators (Table 4). FSWs who reported high levels of collective efficacy were more likely to report CCU with occasional clients than FSWs who reported low levels of collective efficacy (76.7% vs. 71.6%, adjusted OR: 1.3, 95% CI: 1.1–1.7); CCU with regular clients (69.1% vs. 61.9%, adjusted OR: 1.4, 95% CI: 1.1–1.9); STI treatment-seeking from government health facilities (59.8% vs. 32.1%, adjusted OR: 3.3, 95% CI: 2.1–5.1); self-efficacy for condom use (71.4% vs. 63.5%, adjusted OR: 1.5, 95% CI: 1.1–2.0); and self-efficacy for STI service utilization from government health facilities (60.5% vs. 37.3%, adjusted OR: 2.6, 95% CI: 2.1–3.2). Similarly, FSWs who reported high collective action compared to their counterparts were more likely to report CCU with both occasional and regular clients, high self-efficacy for condom use with commercial partners, and high self-efficacy for service utilization from government health facilities.

**Table 4.** Relationship of collectivization with outcome indicators and mediators among female sex workers in Andhra Pradesh (Behavioral Tracking Survey 2010–2011).

	Collective efficacy		Collective agency		Collective action	
Outcome indicators and potential mediators	Low	High	Low	High	Low	High
Outcome indicators						
Consistent condom use with occasional clients (%)	71.6	76.7	76.8	73.8	74.8	79.5
Adjusted OR (95% CI)	Referent	1.3 (1.1–1.7)	Referent	0.9 (0.7–1.1)	Referent	1.3 (1.1–1.8)
Consistent condom use with regular clients (%)	61.9	69.1	69.4	64.7	66.2	73.6
Adjusted OR (95% CI)	Referent	1.4 (1.1–1.9)	Referent	0.8 (0.6–1.1)	Referent	1.5 (1.1–2.0)
STI treatment from government health facilities in past one year (%)	32.1	59.8	49.9	61.9	59.1	44.3
Adjusted OR (95% CI)	Referent	3.3 (2.1–5.1)	Referent	1.6 (1.1–2.2)	Referent	0.5 (0.3–0.8)
Potential mediators						
High self-efficacy for condom use with clients (%)	63.5	71.4	72.0	66.0	68.4	75.7
Adjusted OR (95% CI)	Referent	1.5 (1.1–2.0)	Referent	0.7 (0.5–0.9)	Referent	1.6(1.1–2.2)
High self-efficacy for service utilization from government health facilities (%)	37.3	60.5	48.3	67.7	51.9	72.1
Adjusted OR (95% CI)	Referent	2.6 (2.1–3.2)	Referent	2.1 (1.7–2.6)	Referent	2.4 (1.7–3.5)

Notes: Odds ratios were adjusted for current age (entered as continuous variable); formal schooling (yes, no); marital status (currently married, not currently married); source of income other than sex work (yes, no); place of solicitation for sex work (home, public places, brothel/lodges); visited any place for sex work in past two years (yes, no); duration of sex work in years (entered as continuous variable). OR, odds ratios; CI, confidence intervals; STI, sexually transmitted infection.

Mediation analysis results show significant relationships between FSWs’ high degree of collectivization with outcome indicators in most instances even after adjusting for the effects of the potential mediating factors (Table 6). Only FSWs’ self-efficacy for condom use with commercial partners fully mediated the effects of collective efficacy and collective action on CCU with occasional clients.

### High-risk men who have sex with men

Nearly one-third (31.8%) of the HR-MSM were aged 30 years or more (average age: 27.6 years) and approximately three quarters (74.2%) of them had formal education. About one-third (32.5%) were currently married (Table 3). The majority (79.9%) reported having participated in a public event in the previous six months at risk of being identified as a HR-MSM, while 62.1% reported a high degree of collective efficacy. CCU with paid and paying partners was found to be 69.8% and 72.2%, respectively. Among HR-MSM who suffered from any STI (13.4%) in the past one year, about two-fifths (41.5%) visited a government clinic for treatment.

HR-MSM who participated in any public event compared to those who did not were significantly more likely to report CCU with both paid partners (74.3% vs. 48.1%, adjusted OR: 3.3, 95% CI: 2.1–5.2) and paying partners (75.3% vs. 54.9%, adjusted OR: 2.7, 95% CI: 2.0–3.6), more likely to report high self-efficacy for condom use (74.4% vs. 63.7%, adjusted OR: 1.8, 95% CI: 1.4–2.2), and high self-efficacy for service utilization from government health facilities (59.5% vs. 38.0%, adjusted OR: 2.5, 95% CI: 2.0–3.1). HR-MSM who reported high collective efficacy compared with their counterparts were significantly more likely to use condoms consistently with paying partners, have high self-efficacy for condom use, and high self-efficacy for service utilization from government health facilities (Table 5).

**Table 5.** Relationship of collectivization with outcome indicators and mediators among men who have sex with men/transgenders in Andhra Pradesh (Behavioral Tracking Survey, 2010–2011).

	Participation in public event		Collective efficacy	
Outcome indicators and potential mediators	No	Yes	Low	High
Outcome indicators				
Consistent condom use with paid partners (%)	48.1	74.3	67.9	71.4
Adjusted OR (95% CI)	Referent	3.3 (2.1–5.2)	Referent	1.3 (0.8–1.7)
Consistent condom use with paying partners (%)	54.9	75.3	64.0	76.5
Adjusted OR (95% CI)	Referent	2.7 (2.0–3.6)	Referent	1.9 (1.5–2.3)
STI treatment from government health facilities in past one year (%)	44.9	40.9	42.9	40.5
Adjusted OR (95% CI)	Referent	0.9 (0.4–2.0)	Referent	1.0 (0.6–1.7)
Potential mediators				
High self-efficacy for condom use with clients (%)	63.7	74.4	51.6	84.7
Adjusted OR (95% CI)	Referent	1.8 (1.4–2.2)	Referent	4.9 (4.1–6.0)
High self-efficacy for service utilization from government health facilities (%)	38.0	59.5	35.9	66.9
Adjusted OR (95% CI)	Referent	2.5 (2.0–3.1)	Referent	3.6 (3.0–4.3)

Notes: Odds ratios were adjusted for current age (entered as continuous variable), formal schooling (yes, no), marital status (currently married, not currently married), sex work is main source of income (yes, no), had sex with men/transgenders while visiting any place in past two years (yes, no), duration since first anal sex (entered as continuous variable). OR, odds ratios; CI, confidence intervals; STI, sexually transmitted infection.

Table 6 gives that collectivization continued to have significant positive association with condom use in sex with paid and paying partners even after adjusting for the potential mediating factor.

**Table 6.** Effects of collectivization and mediators on outcome indicators among female sex workers, and high-risk men who have sex with men/transgendres in Andhra Pradesh (Behavioral Tracking Survey 2010–2011).

	FSWs					
	Consistent condom use with occasional clients		Consistent condom use with regular clients		STI treatment from government health facilities in past one year^a^	
Collectivization indicators and corresponding mediators	Adjusted OR for collectivization^b^ (95% CI)	Adjusted OR for mediator^c^ (95% CI)	Adjusted OR for collectivization^b^ (95% CI)	Adjusted OR for mediator^c^ (95% CI)	Adjusted OR for collectivization^b^ (95% CI)	Adjusted OR for mediator^c^ (95% CI)
Collective efficacy and corresponding mediators						
Self-efficacy for condom use with commercial partners	1.1 (0.8–1.5)	2.5 (2.0–3.1)	1.3 (1.1–1.7)	2.3 (1.9–2.8)	–	–
Self-efficacy for service utilization from government health facilities	–	–	–	–	3.6 (2.3–5.6)	0.9 (0.5–1.2)
Collective agency and corresponding mediators						
Self-efficacy for service utilization from government health facilities	–	–	–	–	1.6 (1.2–2.3)	0.7 (0.6–1.0)
Collective action and corresponding mediators						
Self-efficacy for condom use with commercial partners	1.2 (0.9–1.7)	2.7 (2.1–3.5)	1.4 (1.1–1.9)	2.3 (1.9–2.8)	–	–
Self-efficacy for service utilization from government health facilities	–	–	–	–	0.6 (0.4–0.8)	0.8 (0.6–1.1)

	HR-MSM				
	Consistent condom use with paid partners		Consistent condom use with paying partners	
Collectivization indicators and corresponding mediators	Adjusted OR for collectivization^b^ (95% CI)	Adjusted OR for mediator^c^ (95% CI)	Adjusted OR for collectivization^b^ (95% CI)	Adjusted OR for mediator^c^ (95% CI)
Participation in public event and corresponding mediators				
Self-efficacy for condom use with commercial partners	1.9 (1.3–2.9)	2.9 (1.8–4.5)	2.6 (2.0–3.5)	1.6 (1.3–2.1)
Collective efficacy and corresponding mediators				
Self-efficacy for condom use with commercial partners	–	–	1.7 (1.3–2.2)	1.3 (1.1–1.7)

OR, odds ratios; CI, confidence intervals; STI, sexually transmitted infection; FSWs, female sex workers; HR-MSM, High-risk men who have sex with men/transgenders.

a Analysis was restricted to those who reported to have suffered from any STI in past one year.

b Odds ratios were adjusted for the corresponding mediators along with the sociodemographic characteristics: current age (entered as continuous variable), formal schooling (yes, no), marital status (currently married, not currently married), sex work is main source of income (yes, no), had sex with men/transgenders while visiting any place in past two years (yes, no), duration since first anal sex (entered as continuous variable).

c Odds ratios were adjusted for the corresponding collectivization indicator along with the socio-demographic characteristics: current age (entered as continuous variable), formal schooling (yes, no), marital status (currently married, not currently married), sex work is main source of income (yes, no), had sex with men/transgenders while visiting any place in past two years (yes, no), duration since first anal sex (entered as continuous variable).

## Discussion

Community mobilization and structural interventions are widely recognized as complex and characterized by varying approaches that respond to different social, political, and cultural contexts (Beattie et al., 2012; Blanchard et al., 2005; Chakrapani, Newman, & Shunmugam, 2008; Cornish, 2006; Gaikwad et al., 2012; Ghose, Swendeman, George, & Chowdhury, 2008; Gupta, Parkhurst, Ogden, Aggleton, & Mahal, 2008; Wheeler et al., 2012). These approaches led to an array of methodological approaches and theoretical constructs to describe, and measure the community mobilization. The most commonly reported measures of community mobilization include collective efficacy and collective agency, collective action, and participation in community's events. The findings of this research study document a high degree of collective efficacy among four-fifths of FSWs and three-fifths of HR-MSM surveyed in Andhra Pradesh, India. A considerable proportion of FSWs reported collective agency and a large majority of HR-MSM reported participating in public events. These findings are consistent with previous research from other areas where Avahan has implemented community mobilization activities among FSWs (Beattie et al. 2012; Blankenship, Burroway, & Reed, 2010; Gaikwad et al., 2012; T. Thomas et al., 2012).

We found that although large proportions of FSWs reported high collective efficacy and collective agency, relatively few reported high collective action. However, the post hoc analyses suggest that about 43% of FSWs reported that the sex workers group had come together to help FSWs access to at least one of seven entitlements. This result suggests that a greater proportion of sex workers reported the group coming together to help with some entitlements, but not consistently to meet all the needs of FSWs.

Consistent with previous studies (Blankenship et al., 2008; Ghose et al., 2008; Halli et al., 2006; Jana & Singh, 1995; Swendeman, Basu, Das, Jana, & Rotheram-Borus, 2009), the current findings also document that key populations (both FSWs and HR-MSM) reporting a high degree of community collectivization (more specifically, collective efficacy) were more likely to report CCU with various types of partners and STI treatment-seeking behavior from government health facilities. In addition, a high degree of community mobilization had significant positive relationships with self-efficacy for condom use and self-efficacy for service utilization among FSWs and HR-MSM. The study findings add to the current literature in describing the role of potential mediating factors, such as self-efficacy for condom use and self-efficacy for service utilization from government facilities, which might determine the overall effects of collectivization on study outcomes. These associations were independent of sociodemographic characteristics of key populations, which reinforces the importance of community mobilization as a key strategy within HIV prevention interventions in different contexts.

These findings show that positive behavior change is linked with the strong community mobilization among two quite distinct key population groups in India. At the same time, the inconclusive relationship of collectivization with STI treatment-seeking at government health facilities among HR-MSM suggests the need to address structural barriers which prevent HR-MSM from utilizing services at permanent government health centers. Other analysis from India has concluded that stigma and discrimination from health care providers, and poor training to work with HR-MSM (Avahan, 2010; Chakrapani et al., 2008), remain an obstacle.

Although our findings offer important insights into the relationship between community collectivization and selected outcome indicators of an HIV prevention intervention with FSWs and HR-MSM in India, they must be interpreted in the light of certain study limitations. Both the input and outcome indicators were based on self-reports, which are vulnerable to social desirability and recall biases. Most of the outcome variables were based on only one item which may have some validity issues. Furthermore, analyses are cross-sectional and causality cannot be assumed as in the case of prospective research study. Due to the contextual differences in which HR-MSM operate, the collectivization concepts such as collective agency and collection action could not be measured. This to an extent limited our ability to compare the collectivization indicators between these two population groups.

In understanding the findings presented here, the nature of the sample and other inherent biases should be considered. The FSWs and HR-MSM interviewed for the study were drawn primarily from the sites (areas) where Avahan has implemented the community mobilization program. Hence, there was no comparison with areas where no such programs were implemented or across different points of time that could be compared to baseline data. Therefore, findings cannot be generalized to all FSWs and HR- MSM in the national context. However, results could be generalizable to those areas where Avahan or similar intervention settings exist.

Given the widely recognized degree to which contextual factors may impede or facilitate key populations’ access to HIV prevention services and mobilization, more extensive analysis of these factors is needed to inform operational approaches. While a factor analysis was undertaken to correct for biases, the current findings do not offer insights into the influence upon community organizational activities of external environmental factors such as police violence, stigma, legal restrictions, economic power of stakeholders, and control over resources. Among the more critical areas to factor for are the stigma and discrimination faced by HR-MSM and FSWs in Indian society (Chakrapani, Newman, Shunmugam, Kurian, & Dubrow, 2009; Chakrapani, Newman, Shunmugam, McLuckie, & Melwin, 2007; Newman, Chakrapani, Cook, Shunmugam, & Kakinami, 2008; B. Thomas et al., 2012). Further research regarding temporal measures is needed to assess whether, when, and how these factors shape community collectivization.

## Conclusion

The current study contributes to the growing literature on the effects of community mobilization (collective efficacy, collective agency, collective action, and participation in public events) on safe sex behavior and STI service utilization from government health facilities in India by examining these issues among those at greater risk for acquisition and transmission of HIV – FSWs and HR-MSM. The findings document that community collectivization is associated with CCU and negotiation for condom use. These data highlight the need for programs to integrate community mobilization approaches as part of HIV prevention, in India and elsewhere. Perhaps most importantly, the findings emphasize the value of collectivizing key populations to address the situations/crises they face. Globally, structural intervention approaches are considered critical to HIV prevention (Hecht et al., 2010) but recognizing the process of collectivization as a prerequisite for addressing safe sex behavior and health service utilization may offer greater opportunities to address the stigma and discrimination experienced by marginalized groups (Chakrapani et al., 2007, 2009; Newman et al., 2008; B. Thomas et al., 2012). While the current study findings offer evidence that high-risk behaviors are mitigated in mobilized populations, many individuals within the study population still report low levels of collective action, showing that more work is needed to support communities in the Avahan program, especially considering that program implementation is due to be handed over to the Government of India by 2013. As all HIV prevention efforts in India – including Avahan – are being transitioned to the government's National AIDS Control Program, such an initiative calls for further strengthening and sustaining community mobilization approaches as part of a multi-faceted effort to ensure strong service utilization and safe sex behaviors.
